# Multiple metachronous foveolar-type gastric adenomas in a *Helicobacter pylori*-naïve patient with long-term use of a proton pump inhibitor: a case report

**DOI:** 10.1007/s10120-025-01595-w

**Published:** 2025-02-13

**Authors:** Yoichi Miyaoka, Kotaro Shibagaki, Ryoji Kushima, Taisuke Omachi, Takanobu Hino, Aya Fujiwara, Kousuke Tsukano, Sayaka Ogawa, Satoshi Yamanouchi, Masaki Tanaka, Tatsuya Miyake, Hirofumi Fujishiro, Naruaki Kohge, Hideyuki Ohnuma, Norihisa Ishimura, Tsuyoshi Mishiro, Shunji Ishihara

**Affiliations:** 1 Department of Endoscopy, Shimane Prefectural Central Hospital, Izumo, Japan; 2 Department of Endoscopy, Shimane University Hospital, 89-1 Enya, Izumo, 693-8501 Japan; 3 Department of Pathology, Shiga University of Medical Science, Otsu, Japan; 4 Department of Gastroenterology, Shimane Prefectural Central Hospital, Izumo, Japan; 5 Department of Hepatology, Shimane Prefectural Central Hospital, Izumo, Japan; 6 Department of Pathology, Shimane Prefectural Central Hospital, Izumo, Japan; 7 Department of Gastroenterology, Shimane University Hospital, Izumo, Japan

**Keywords:** *Helicobacter pylori*, Foveolar-type gastric adenoma, Proton pump inhibitor

## Abstract

A 69-year-old man undergoing long-term administration of a proton-pump inhibitor (PPI) underwent upper endoscopy, which found a small, whitish, flat lesion in the fundic gland (oxyntic) mucosa. The patient had never received treatment for *Helicobacter pylori* (*Hp*) infection, and diagnostic testing for *Hp* was negative, suggesting an *Hp*-naïve status. Two years later, the lesion appeared markedly enlarged and was endoscopically resected. Histological examination revealed a low-grade foveolar-type gastric adenoma (FGA), predominantly expressing MUC5AC by immunohistochemistry. Two years later, while PPI therapy was continued, three new flat lesions were found. These were endoscopically resected and histologically diagnosed as low-grade FGAs as before, suggesting that multiple metachronous tumors had developed in a short period of time during long-term PPI administration. A *KRAS* mutation and a *CTNNB1* mutation were identified in the tumor. To our best knowledge, this is the first report of potentially PPI-associated multiple metachronous FGAs in an *Hp*-naïve patient. Here we report a case of multiple foveolar-type gastric adenomas with rapid metachronous recurrences during long-term use of a proton pump inhibitor in *Helicobacter pylori*-naïve patient.

**Supplementary Information:**

The online version contains supplementary material available at 10.1007/s10120-025-01595-w.

## Introduction

Chronic infection with *Helicobacter pylori* (*Hp*) is a major risk factor for gastric cancer [1]. However, the recent decline in *Hp* infection rates in Japan has led to an increase in reports of gastric neoplasms in *Hp*-naïve individuals [2]. Among these *Hp*-naïve gastric neoplasms (HpNGNs), foveolar-type gastric adenoma (FGA), and fundic gland polyp with dysplasia (FGPD) both show foveolar cell differentiation. Both are classified into syndromic and sporadic types, with syndromic types occurring in patients with familial adenomatous polyposis or gastric adenocarcinoma with proximal gastric polyposis [3, 4]. Sporadic FGA is morphologically divided into the flat type and the raspberry type, which recent genomic studies have also shown to exhibit distinct genetic profiles [5, 6]. The raspberry type appears as a small reddish lesion and represents the majority of sporadic FGA [7, 8]. The flat type shares several histological and genetic features with syndromic FGA, except for their different clinical backgrounds [9]. FGPD presents as neoplastic foveolar epithelium in the superficial layer of the FGP, and sporadic type mostly occurs in patients with chronic use of proton pump inhibitors (PPIs) [10, 11].

Here we report a case of multiple sporadic flat-type FGAs with rapid metachronous recurrences during long-term use of a PPI.

## Case report

**Fig. 1:**
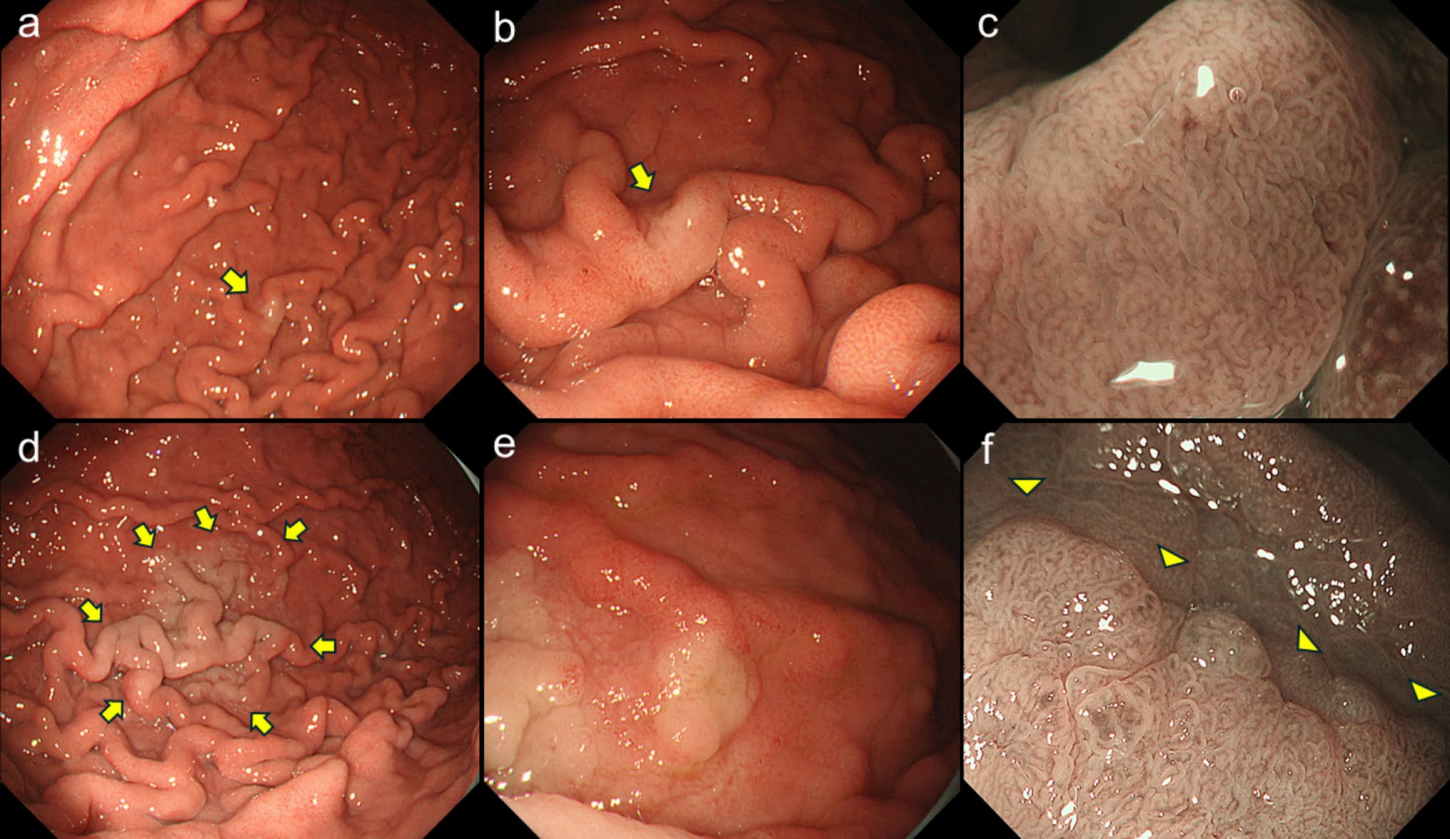
Changes in endoscopic findings over time (lesion A). A small flat whitish lesion was observed in the non-atrophic fundic gland mucosa (arrow) (**a, b**). Narrow band imaging with magnification endoscopy shows an irregularly shaped tubular structure (**c**). Two years later, the lesion was significantly enlarged (arrows, **d**). The lesion showed a course, flatly elevated shape (**e**) with a papillary-dominant superficial microstructure clearly demarcated from the adjacent mucosa (arrowheads, **f**), suggesting an epithelial neoplasm

A 69-year-old man with a 3-year history of continuous PPI use (esomeprazole 20 mg daily) for Barrett’s esophagus. The patient underwent a follow-up upper endoscopy, which revealed a small whitish flat lesion on the non-atrophic fundic gland mucosa (lesion A) (Fig. 1a–c). Two years later, the lesion was markedly enlarged, and narrow-band imaging with magnifying endoscopy showed an irregular superficial microstructure that was clearly demarcated from the adjacent mucosa (Fig. 1d–f). A biopsy specimen showed histological evidence of low-grade foveolar-type gastric dysplasia.

The patient had never received *Hp* eradication therapy. His serum anti-*Hp* IgG antibody titer was < 3 U/mL, and a ^13^C-labeled urea breath test value was < 2.5‰, findings consistent with *Hp*-naïve status. The serum gastrin level was 1960 pg/mL, indicating hypergastrinemia due to continuous PPI use. As the endoscopic features of the lesion were similar to flat-type FGA, a colonoscopy was performed to rule out a syndromic FGA, but only a few colonic adenomas were found. There was no family history of familial adenomatous polyposis or gastric adenocarcinoma with proximal polyposis of the stomach. The tumor was therefore considered to be a sporadic tumor arising from *Hp*-naïve gastric mucosa. It was completely resected by endoscopic submucosal dissection.

**Fig. 2:**
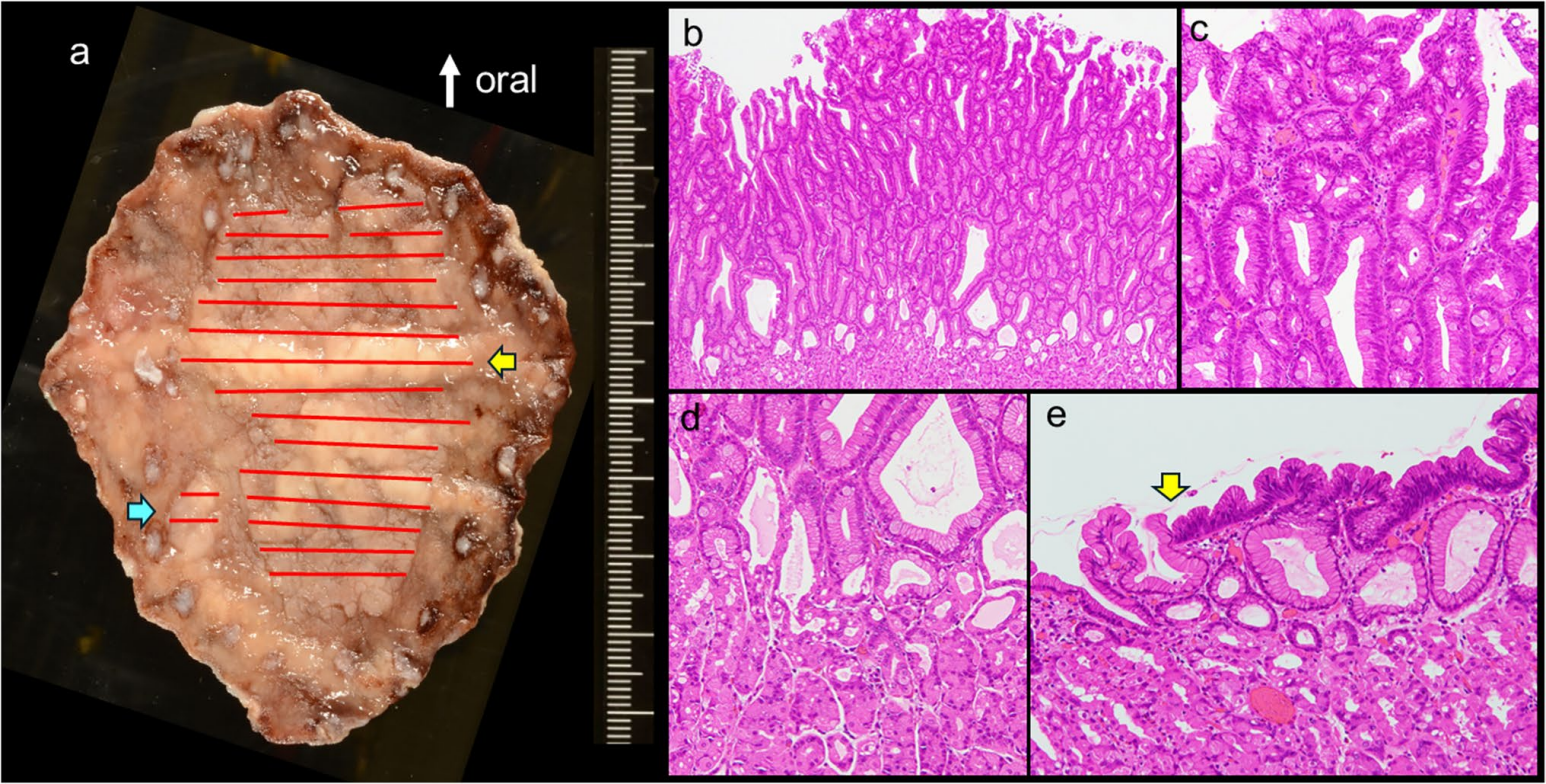
Resected specimen and histologic findings (lesion A). The resected lesion measured 67 × 48 mm (yellow arrow), with a small satellite lesion (blue arrow) (**a**). The lesion consisted mainly of atypical tall columnar cells resembling gastric-foveolar cells with tubular-dominant and superficial papillary structures (**b**). The nuclei, round to oval and slightly elongated in shape, were basically located (**c**). The fundic gland tissue was preserved under the neoplastic glands (**d**). In the periphery, the neoplastic epithelium showed thin, superficially spreading growth with a boundary distinct from the adjacent foveolar epithelium (arrow), suggesting a low-grade FGA. The fundic gland tissue showed parietal cell enlargement with protrusion into the lumen of the gland, suggesting a PPI-associated gastropathy (**e**)

The resected specimen showed a 67 × 48 mm lesion with a small satellite lesion (Fig. 2a). The lesion consisted mainly of atypical tall columnar cells resembling gastric-foveolar cells with tubular-dominant and superficial papillary structures (Fig. 2b). The nuclei, round to oval and slightly elongated in shape, were basically located (Fig. 2c). In the deep and peripheral areas of the lesion, there was a clear border with the normal gastric fundic gland mucosa epithelium (Fig. 2d–e). Consequently, the lesion was diagnosed as a low-grade FGA according to the WHO classification [3, 12].

**Fig. 3:**
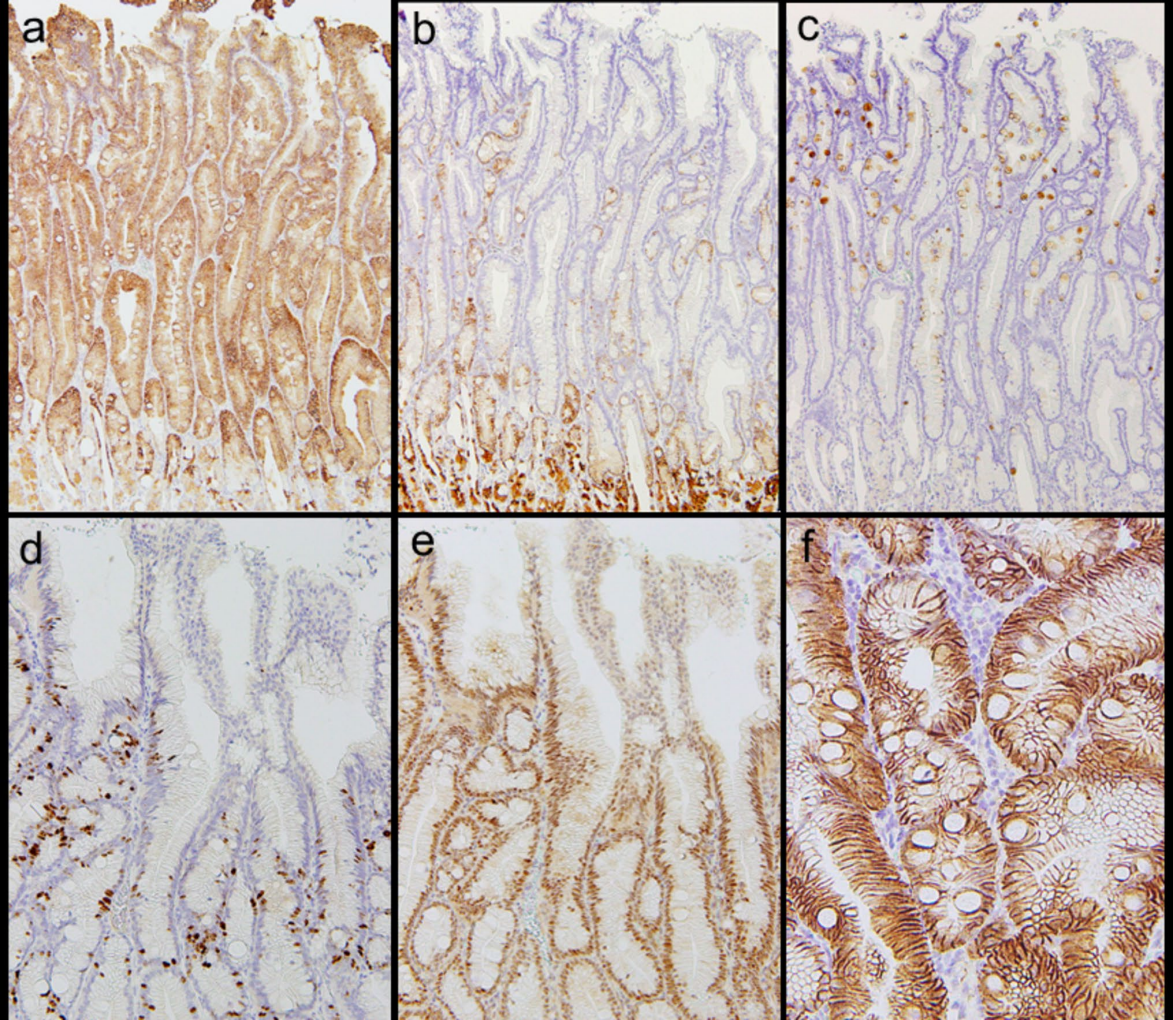
Immunohistochemical analysis (lesion A). MUC5AC was strongly, diffusely, expressed in the neoplastic cells (**a**), MUC6 was partly expressed in the lower layer of the neoplasm (**b**), and MUC2 was sporadically expressed in the neoplasm (**c**), exhibiting a gastric-dominant immunophenotype (foveolar cell differentiation). Ki-67 (**d**) and p53 (**e**) were diffusely expressed in the lower to middle layers of the neoplasm, and the expression was reduced in the superficial layer. β-catenin was expressed in the cell membranes and cytoplasm, but its nuclear translocation is not seen (**f**)

Immunohistochemical analysis showed strong diffuse MUC5AC expression in neoplastic cells (Fig. 3a), partial MUC6 expression in the lower neoplasm layer (Fig. 3b), and sporadic MUC2 expression in the neoplastic epithelium (Fig. 3c), with no intestinal metaplasia in the surrounding epithelium. Other intestinal markers (CD10 and CDX2) were negative, supporting foveolar cell-dominant differentiation. Ki-67 and p53 were diffusely expressed in the lower to middle layers of the neoplasm, with reduced expression in the superficial layer (Fig. 3d, e). β-catenin was expressed in the cell membranes and cytoplasm, whereas its nuclear translocation was not confirmed (Fig. 3f). These findings were consistent with those of gastric foveolar-type dysplasia and adenoma.

**Fig. 4:**
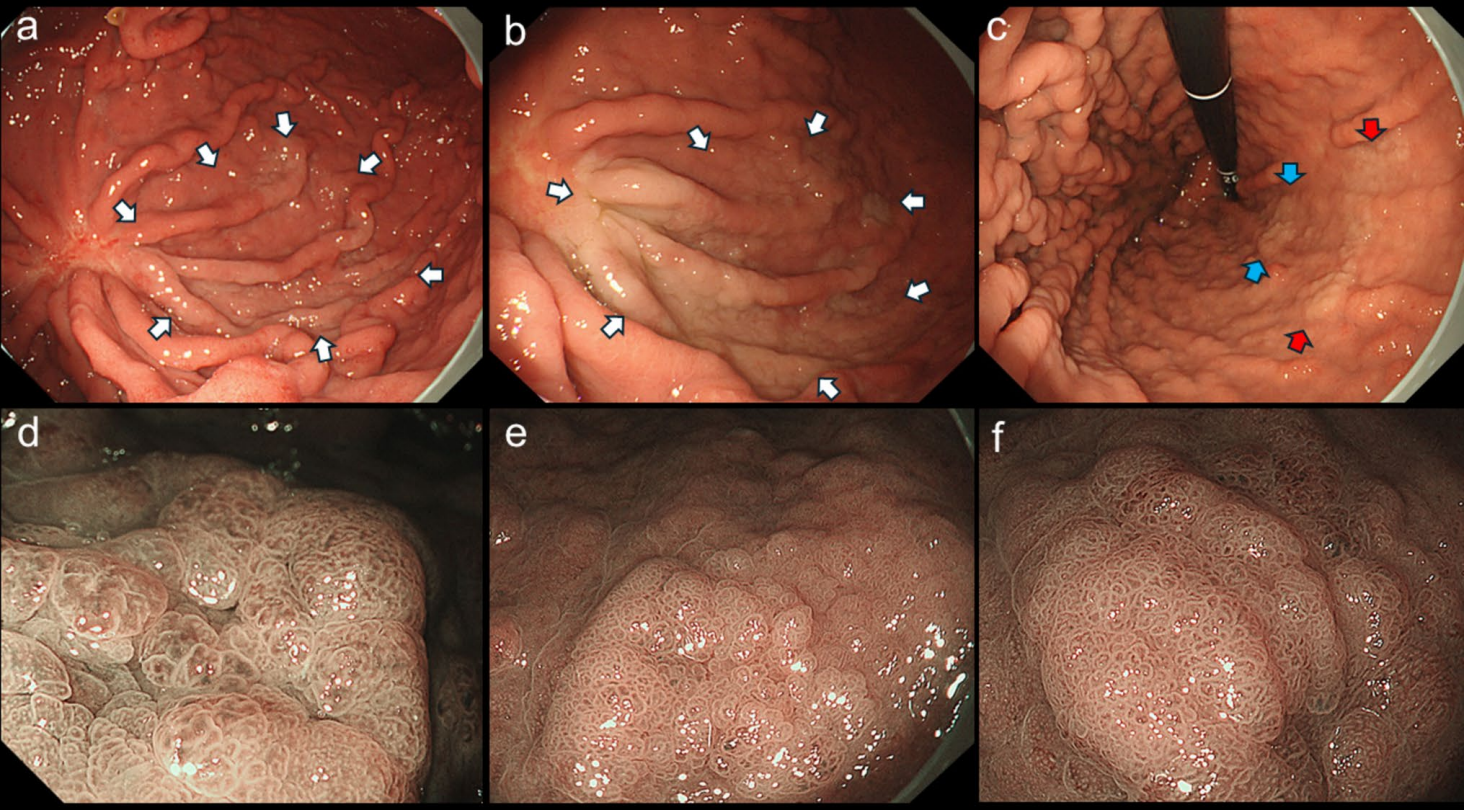
Changes in the endoscopic findings over time (lesions B, C, and D). Two months after the endoscopic resection of lesion A, an opaque flat lesion was newly identified near the scar of the resected specimen (lesion B, white arrows, **a**). Two years later, lesion B had thickened and was more clearly visualized (white arrows, **b**), and two more lesions had appeared de novo at the lesser curvature of the gastric body (lesion C, blue arrows; lesion D, red arrows, **c**). Narrow-band imaging with magnification endoscopy of each lesion showed irregularly shaped, papillary-dominant superficial microstructures (d, lesion B; e, lesion C; f, lesion D), suggesting multiple metachronous recurrences

The patient was continuously treated with a PPI after endoscopic treatment. Two months later, a follow-up endoscopy revealed a new flat whitish lesion (lesion B) with indistinct contours near the initially resected lesion (Fig. 4a). Two more years later, lesion B was clearly demarcated from the surrounding mucosa (Fig. 4b), and two new lesions were detected in the lesser curvature of the gastric body (lesions C and D, Fig. 4c). Narrow-band imaging with magnification endoscopy showed an irregular superficial microstructure, suggesting neoplastic characteristics (Fig. 4d–f). Biopsy specimens from each lesion showed histologic evidence of low-grade foveolar-type gastric dysplasia.

**Fig. 5:**
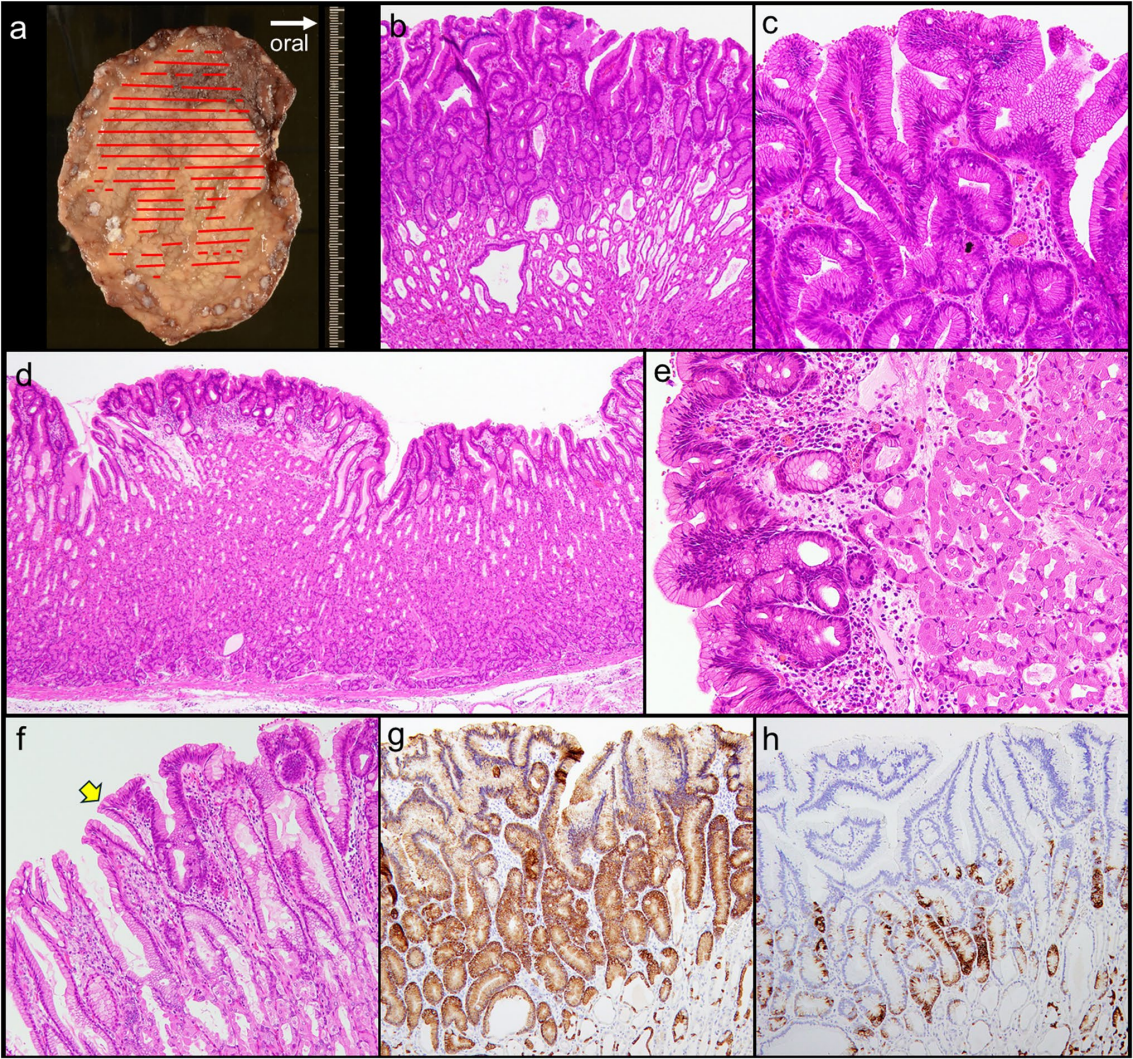
Resected specimen and histologic findings (lesion B). The resected lesion measured 97 × 76 mm and was completely resected (**a**). Neoplastic tissue was seen growing over the non-atrophic fundic glands that show cystic dilation (**b**), and the findings were diagnosed as a low-grade FGA (**c**). The lesion exhibits lateral spreading growth of neoplastic glands in a superficial layer across the lamina propria mucosae, which is thickened by hyperplastic changes in parietal cells (**d**), characterized by cellular enlargement and protrusion into the dilated glandular lumen (**e**), consistent with PPI-associated gastropathy. In the periphery, the neoplastic epithelium showed thin, superficially spreading growth with a boundary distinct from adjacent foveolar epithelium (arrow, **f**). MUC5AC was diffusely expressed in the neoplastic cells (**g**), and MUC6 was partly expressed in the lower layer of the neoplasm (**h**), as seen in lesion A

All three lesions were resected endoscopically. Histologically, they were all diagnosed as low-grade FGAs, which were consistent with the findings of the first resected lesion. Lesion B was 97 × 76 mm (Fig. 5), lesion C was 26 × 23 mm (Supplementary Figs. 1a-1d), and lesion D was 50 × 15 mm (Supplementary Fig. S1e–h). The lamina propria mucosae is thickened due to hyperplastic changes in parietal cells, characterized by cellular enlargement and protrusion into the dilated glandular lumen, consistent with PPI-associated gastropathy. The whole-exome sequencing was performed on lesion B with the approval of the hospital’s ethics committee and the patient's informed consent, comparing neoplastic and normal mucosa using the NovaSeq system. Variants with a variant allele frequency (VAF) greater than 10% were classified as mutations. A *KRAS* mutation (p.G13C) and a *CTNNB1* mutation (p.Leu366Phe) were identified, with VAFs of 43.5% and 10.1%, respectively. No mutations were identified in the *APC* or *TP53* genes.

After endoscopic resection, PPI treatment was adjusted to on-demand use to reduce the potential risk of tumorigenesis due to chronic PPI use. Serum gastrin levels normalized, and periodic follow-up endoscopy showed improvement in wall thickening and fold meandering, while histological findings remained largely unchanged (Supplementary Fig. S2). The patient has been undergoing regular follow-up endoscopy, with no evidence of metachronous recurrence observed over the past 4 years.

## Discussion

The lesions in our patient were *Hp*-naïve FGAs with rapid growth and metachronous recurrence over a short period of time during chronic PPI treatment. Given the clinical course, the possibility of non-neoplastic features should be considered. However, the histology of the lesions showed laterally spreading growth of atypical foveolar cells with boundaries distinct from the adjacent epithelium, suggesting multiple low-grade FGAs.

HpNGNs with foveolar cell-dominant differentiation include FGA and FGPD. Sporadic flat-type FGA and FGPD usually present as a solitary lesion with slow growth, the latter mostly occurring in patients on long-term PPI treatment [10, 11, 13]. Our patient's lesions resembled flat-type FGA in terms of endoscopic findings and direction of cellular differentiation, but some clinicopathological features were closer to FGPD. Histologically, the lesions consisted of tubular-dominant structures with superficially spreading growth in the periphery, as seen in the histological findings of FGPD [10, 11], whereas flat-type FGAs primarily show an elongated papillary structure [3, 9]. Immunohistochemical analysis revealed that the lesions showed a MUC5AC-dominant immunophenotype (foveolar cell differentiation). Sporadic MUC2 expression is typically absent in FGA and FGPD but has been occasionally reported in foveolar-type dysplasia [12, 14]. CDX2 promotes MUC2 expression but is not always co-expressed [15]; CDX2-negative/MUC2-positive gastric cancers have been reported [16, 17], indicating this finding can occur in MUC2-positive gastric tumors. Nuclear translocation of β-catenin, observed in most flat-type FGAs and some FGPDs with APC mutations [9–11], was absent in the lesions, likely due to the absence of an APC mutation. While the tumor exhibited incomplete characteristics of both flat-type FGA and FGPD, it was classified as an FGA based on the WHO diagnostic criteria [3].

Naka et al. reported that all sporadic flat-type FGAs harbor both *KRAS* and *APC* mutations, with *KRAS* mutations predominantly located at G12 and the remainder at Q61 [9]. In this case, no mutations were identified in the *APC* gene; however, a *KRAS* mutation (p.G13C) was detected, indicating a potential role in tumor pathogenesis due to its high VAF. Similarly, Abraham et al. noted that over half of sporadic FGPDs harbor mutations in *APC* and/or *CTNNB1* [18], and in this case, a *CTNNB1* mutation (p.Leu366Phe) was identified. Notably, this tumor exhibited genomic alterations and clinicopathological features that overlapped with both flat-type FGAs and FGPDs, despite the absence of an *APC* mutation. Ki-67 and p53 expression were diffusely observed throughout the neoplasm, with reduced expression in the superficial layer. This pattern may reflect suppressed p53 protein degradation driven by metabolic abnormalities, independent of *TP53* mutations in proliferating cells.

To our knowledge, this is the first report of potentially PPI-associated multiple FGAs in an *Hp*-naïve patient. With the recent increase in the use of PPIs, including potassium-competitive acid blockers, so-called “PPI-associated gastropathy” has become more common in daily endoscopic practice. Chronic use of PPIs increases the proliferation of gastric epithelial cells due to hypergastrinemia, resulting in enlarged parietal cells protruding into the lumen of the glands, cystic dilatation of the fundic glands, and foveolar hyperplasia [19, 20]. In addition, several observational studies suggest that long-term PPI use is also associated with an increased risk of gastric carcinoma [21, 22]. The association with HpNGNs has not been clarified, but most patients with FGPD (79%─82%) have been reported to have used PPIs [10, 11].

In conclusion, long-term use of PPIs in *Hp*-naïve individuals may cause foveolar-type gastric dysplasia/adenoma not only on FGP but also on morphologically normal gastric fundic gland mucosa. Further studies are needed to evaluate the potential role of prolonged PPI use in tumorigenesis among *Hp*-naïve patients.

## Supplementary Information

Below is the link to the electronic supplementary material.

Supplementary file1 (TIF 2214 kb)

Supplementary file2 (TIF 1994 kb)

## Abbreviations

FGA: Foveolar-type gastric adenoma
FGP: Fundic gland polyp
FGPD: Fundic gland polyp with dysplasia
Hp: *Helicobacter pylori*
HpNGN: *Helicobacter pylori*-Naïve gastric neoplasm
PPI: Proton pump inhibitor
VAF: Variant allele frequency
