# Supplementary figures and images for: Multiple metachronous foveolar-type gastric adenomas in a Helicobacter pylori-naïve patient with long-term use of a proton pump inhibitor: a case report
Source: Gastric Cancer. 2025 Feb 13;28(3):537–43. doi: 10.1007/s10120-025-01595-w (PMC11993507; doi:10.1007/s10120-025-01595-w)

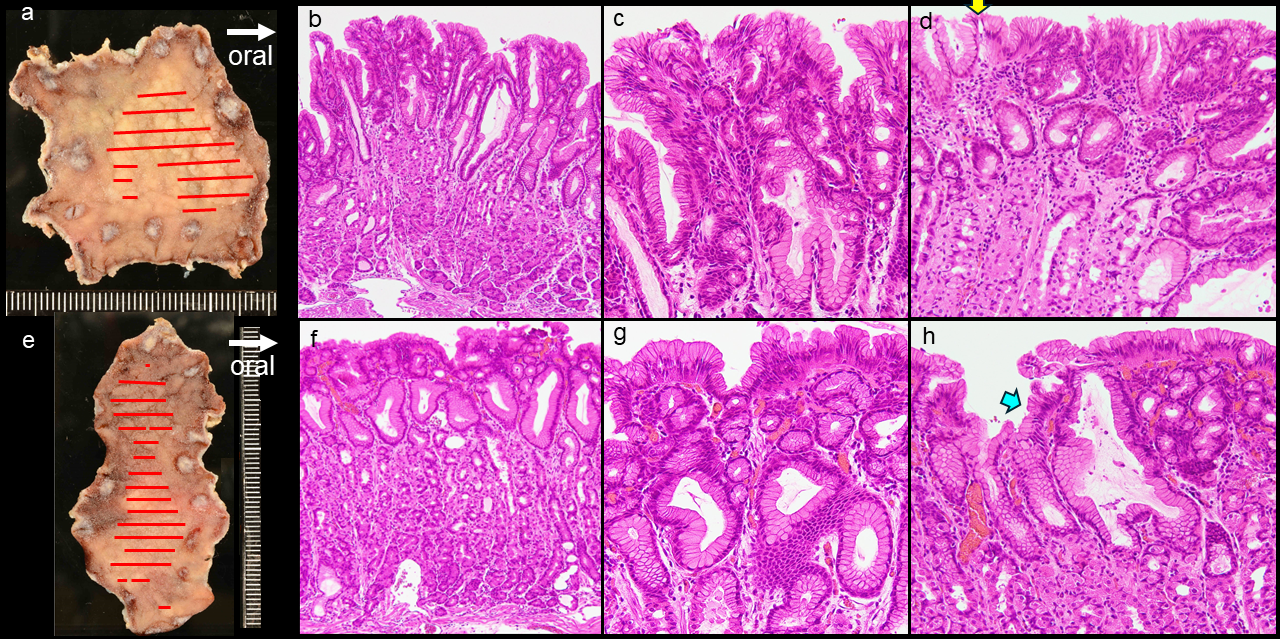

Supplement: Supplementary file 1 — Supplementary file1 (TIF 2214 kb) [file 10120_2025_1595_MOESM1_ESM.tif]

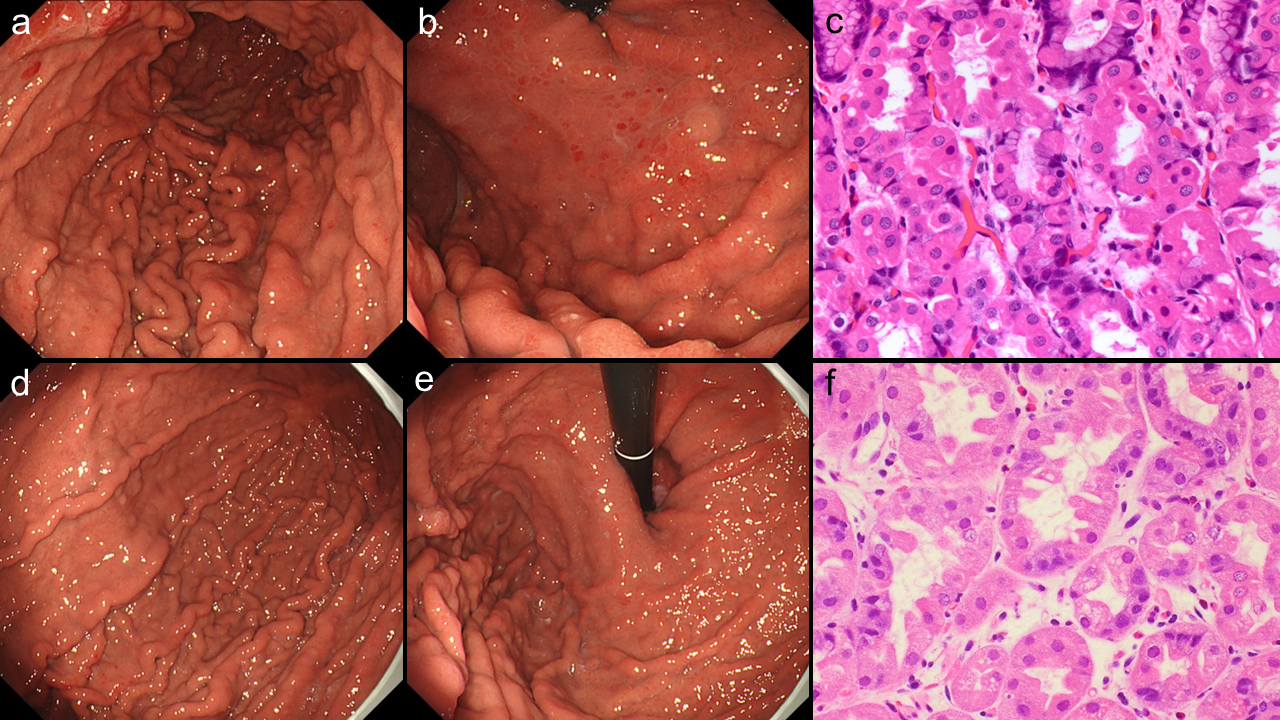

Supplement: Supplementary file 2 — Supplementary file2 (TIF 1994 kb) [file 10120_2025_1595_MOESM2_ESM.tif]
